# A Hybrid Brain-Computer Interface Based on Visual Evoked Potential and Pupillary Response

**DOI:** 10.3389/fnhum.2022.834959

**Published:** 2022-02-03

**Authors:** Lu Jiang, Xiaoyang Li, Weihua Pei, Xiaorong Gao, Yijun Wang

**Affiliations:** ^1^State Key Laboratory on Integrated Optoelectronics, Institute of Semiconductors, Chinese Academy of Sciences, Beijing, China; ^2^College of Materials Science and Opto-Electronic Technology, University of Chinese Academy of Sciences, Beijing, China; ^3^Department of Biomedical Engineering, School of Medicine, Tsinghua University, Beijing, China; ^4^School of Future Technology, University of Chinese Academy of Sciences, Beijing, China; ^5^Chinese Institute for Brain Research, Beijing, China

**Keywords:** hybrid brain-computer interface, electroencephalogram, visual evoked potential, pupillary response, BCI illiteracy, task-related component analysis, canonical correlation analysis

## Abstract

Brain-computer interface (BCI) based on steady-state visual evoked potential (SSVEP) has been widely studied due to the high information transfer rate (ITR), little user training, and wide subject applicability. However, there are also disadvantages such as visual discomfort and “BCI illiteracy.” To address these problems, this study proposes to use low-frequency stimulations (12 classes, 0.8–2.12 Hz with an interval of 0.12 Hz), which can simultaneously elicit visual evoked potential (VEP) and pupillary response (PR) to construct a hybrid BCI (h-BCI) system. Classification accuracy was calculated using supervised and unsupervised methods, respectively, and the hybrid accuracy was obtained using a decision fusion method to combine the information of VEP and PR. Online experimental results from 10 subjects showed that the averaged accuracy was 94.90 ± 2.34% (data length 1.5 s) for the supervised method and 91.88 ± 3.68% (data length 4 s) for the unsupervised method, which correspond to the ITR of 64.35 ± 3.07 bits/min (bpm) and 33.19 ± 2.38 bpm, respectively. Notably, the hybrid method achieved higher accuracy and ITR than that of VEP and PR for most subjects, especially for the short data length. Together with the subjects’ feedback on user experience, these results indicate that the proposed h-BCI with the low-frequency stimulation paradigm is more comfortable and favorable than the traditional SSVEP-BCI paradigm using the alpha frequency range.

## Introduction

Brain-computer interface (BCI) allows people to establish an alternative communication channel between the user’s intention and output devices which is completely independent of the normal motor output paths of the nervous system (Gandhi, 2007; Volosyak et al., 2017). BCI is especially relevant for severely disabled users, such as amyotrophic lateral sclerosis, spinal cord injury, and stroke victims (Hoffmann et al., 2008; Kuebier et al., 2009), while applications in entertainment, safety, and security are also emerging (Zhu et al., 2010). Electroencephalography (EEG) is the most favorable method in non-invasive BCIs (Gao et al., 2014) due to its essential attributes such as low cost, high time resolution, and easy access to data (Mason et al., 2007; Volosyak, 2011).

Many laboratories and clinical tests have demonstrated the convincing robustness of visual evoked potential (VEP)-based BCI systems (Wang et al., 2008). In the 1970s, Vidal developed a BCI system that used VEPs to determine the visual fixation point (Vidal, 1977). Among different types of VEPs, steady-state visual evoked potential (SSVEP) is a continuous electrical activity recorded at the occipital and parietal cortex areas, which is elicited at the same frequency (and/or harmonics) when the retina is excited by visual stimuli at a specific frequency (Luo and Sullivan, 2010). The stimulation frequency can be divided into low (below 12 Hz), medium (12–30 Hz), and high (above 30 Hz) frequency bands (Bouma, 1962; Floriano et al., 2019). SSVEP has been recognized as a reliable, fast, and easy-to-use communication paradigm (Allison et al., 2010) due to its high information transfer rate (ITR), little training cost, and fewer electrode requirements (Hoffmann et al., 2009; Brunner et al., 2010).

Nevertheless, SSVEP-BCI also has some disadvantages that need to be improved, such as fatigue, discomfort, safety, and user limitation. First of all, durative visual stimulations can cause dizziness and visual fatigue, even impair the user’s vision, and the amplitude of SSVEP response will also be reduced (Wu and Su, 2014). Secondly, the alpha frequency range (8–13 Hz) used in the traditional SSVEP paradigm offers low level of comfort (Zhu et al., 2010). Our pre-experiment on the effect of stimulation frequency on user experience has found that the flickering stimulations in the low-frequency range (<3 Hz) can provide a better comfort level to the alpha frequency range. Stimulation frequency higher than the critical fusion frequency (CFF, e.g., 60 Hz) makes people feel comfortable with imperceptible flickers (Hartmann et al., 1978; Williams et al., 2004). Frequency-modulated (FM) and amplitude-modulated (AM) methods (Chang et al., 2014; Dreyer and Herrmann, 2015; Dreyer et al., 2017) have been adopted to reduce visual fatigue and enhance the user experience of the SSVEP-BCI in the alpha frequency band. Thirdly, the flickering stimulus has some possibility of causing seizures in photosensitive individuals. Photosensitivity is an abnormal brain electrical response to light or pattern stimulation, which occurs in 0.3–3% of the population (Fisher et al., 2005). Harding investigated the proportion of photoparoxysmal responses in 170 photosensitive patients (Harding and Harding, 2010). The results indicated that only 3% of photosensitive patients were at risk at 3 Hz, compared with a maximum of 90% at 16 Hz. Finally, “BCI illiteracy” is a common problem in the BCI field. A non-negligible portion of the subjects (estimated from 15 to 30%) were unable to achieve control of the interface because they did not show the expected brain activity modulated by the mental task (Vidaurre and Blankertz, 2009). Up to now, there is still no universal BCI applicable to all users. For example, Brunner asked 14 healthy subjects to complete a visual attention task to produce SSVEP and an imagined movement task to produce event-related desynchronization (ERD) (Brunner et al., 2010). The number of illiterates was 11 in the ERD condition and was 3 in the SSVEP condition. In order to improve the accuracy of users with poor performance, they completed an offline simulation of a hybrid BCI (h-BCI) in which the subjects performed the two tasks simultaneously, and the number of illiterates was reduced to one in the hybrid condition. To improve user experience of VEP-BCIs, this study proposes to design and implement a hybrid BCI system based on the low-frequency (<3 Hz) stimuli with high comfort level and safety.

Hybrid BCI can improve the classification accuracy, increase the number of commands, and shorten the detection time of the BCI system by combining two or more patterns (at least one of which is a brain signal) (Hong and Jawad, 2017). Recently, pupillary responses (PR), such as the pupillary light reflex, have been used as the second pattern in addition to EEG due to the low user burden, non-invasiveness, and no need for training (Muto et al., 2020). Pupil diameter changes steadily with the illuminance of the observed object to regulate the amount of light entering the eye (Crawford, 1936; Woodhouse, 1975; Woodhouse and Campbell, 1975), and the modulation frequency of PR is synchronized with the luminance-modulation frequency of the visual stimulus. The amplitude of PR decreases as the stimulation frequency increases (Muto et al., 2020), and the consistent, measurable PR can be induced at the flickering frequency up to 2.3 Hz (Naber et al., 2013). Compared with the detection of gaze position (Ma et al., 2018; Yao et al., 2018), the measurement of PR does not require system calibration. There were only few studies on human-computer interaction (HCI) based on PR. Sebastiaan presented a human-computer interface based on decoding of attention through pupillometry (Sebastiaan et al., 2016). Two sets of items with the same flickering frequency (0.8 Hz) and opposite phase were presented on the display, and each participant covertly attended to one set, and the pupil size reflected the illuminance of the selected items. The binary classification was realized based on PR, and the mean accuracy of ten subjects was 88.9%, resulting in an ITR of 2.58 bits/min (bpm) with a mean selection time of 14.9 s. Ponzio et al. (2019) proved that the illuminance was the only factor that significantly affects pupil constriction and found no differences between the monocular and the binocular vision. They also established a binary communication based on PR and achieved an accuracy of 100% at 10 bpm and 96% at 15 bpm. Muto et al. (2020) realized an information input interface with 12 options (from 0.58 to 1.90 Hz, with an interval of 0.12 Hz) based on PR. The averaged power spectral density (PSD) peak decreased with increasing luminance-modulation frequency, and the averaged classification accuracy reached 85.4% with a data length of 7 s. PR and SSVEP have also been combined to implement an h-BCI. De’Sperati et al. (2020) reported a frequency tagging approach based on the evoked oscillatory responses of the pupil and the visual cortex. Each of the two flickers contained a sum of two sinusoidally modulated luminance (0.9 and 15 Hz for one stimulus, 1.4 and 20 Hz for another stimulus). By applying a binary linear classifier to PR and SSVEP signals for 18 subjects, hybrid classification accuracy was 83% with the data length of 7.5 s, which was higher than that of PR (75%) and SSVEP (80%). These PR-based HCI and h-BCI studies showed limitations, such as the small number of targets and the longer detection time compared with the existing SSVEP-BCIs (Nakanishi et al., 2018; Mao et al., 2020; Ming et al., 2021).

This study intends to use low-frequency visual stimulations that can simultaneously elicit VEP and PR (Jiang et al., 2020) to implement a 12-target h-BCI speller. Compared with the existing HCI and BCI work related to PR, this system aims to achieve a shorter detection time and higher classification accuracy by adopting efficient coding and decoding methods. Compared to other VEP-based BCIs, the proposed system has the advantage of better comfort and is applicable to more subjects.

## Materials and Methods

### Experimental Environment

#### Subjects

Ten healthy subjects (2 males and 8 females, ages 23–29 years, with a mean age of 25.7) participated in the offline and online BCI experiments. Twelve subjects (6 males and 6 females, ages 23–28 years, with a mean age of 25) participated in the behavioral test of user experience without EEG or PR recording, and 5 of them participated in the BCI experiments. All subjects had normal or corrected-to-normal vision by wearing contact lenses. Before the experiment, each subject was asked to read and sign an informed consent form approved by the Research Ethics Committee of Tsinghua University.

#### Data Recording

In the experiment, EEG data were recorded by a SynAmps2 amplifier (Neuroscan Inc.) at a sampling rate of 1,000 Hz. According to the international 10–20 system, nine electrodes (Pz, PO5, PO3, POz, PO4, PO6, O1, Oz, and O2) were placed at the occipital and parietal regions to record VEPs, and two electrodes located on the forehead (AFz) and vertex (between Cz and CPz) regions as ground and reference, respectively. The contact impedance was kept below 10 kΩ. The binocular PR data were recorded by an infrared eye tracker (EyeLink 1000 Plus, SR Research Inc.) with a sampling rate of 1,000 Hz, and the PR data were preprocessed by an interpolation method (Kret and Sjak-Shie, 2019). The focal length of the lens was adjusted manually to observe a clear pupil image and stable corneal reflection point.

#### Visual Stimulus Design

**FIGURE 1:**
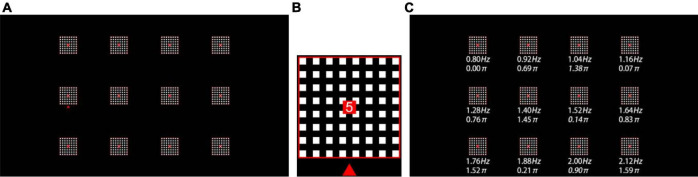
**(A)** Stimulation interface of the 12-target BCI. **(B)** Stimulation pattern (e.g., target 5) with fixation point (a red square), character and visual cue (a red triangle). **(C)** Frequency and phase values for all targets.

This study designed a 12-target online h-BCI system to realize a virtual keypad. A 24.5-inch LCD display (Dell AW2518H) with a resolution of 1280 × 720 pixels and a refresh rate of 240 Hz was used to present a 3 × 4 stimulus matrix (Figure 1A). The size of each stimulus was 75 × 75 pixels (3° × 3°), and the horizontal and vertical distances between the centers of any two adjacent stimuli were 225 pixels (9°). Each target adopted the style of a grid stimulus (Ming et al., 2021), which consisted of 8 × 8 small flickering squares. The size of each square was 5 × 5 pixels. The 12 stimulus targets correspond to 12 characters (1, 2, 3, 4, 5, 6, 7, 8, *, 9, 0, and #). As shown in Figure 1B, a red square with a size of 10 × 10 pixels in the center of each stimulus was used to highlight the characters and serve as the subject’s fixation point. The stimulation was drawn and stably presented by the Psychophysics Toolbox Ver. 3 (Brainard, 1997) in MATLAB (MathWorks, Inc), as evidenced by the high consistency of visual stimulation waveforms in multiple trials recorded by a photodiode.

All visual stimuli were coded according to the joint frequency-phase modulation method (Chen et al., 2015b), as shown in Figure 1C. Twelve frequencies (from 0.8 to 2.12 Hz, with 0.12 Hz interval) were used, and the phase difference between two adjacent frequencies was set to 0.69π in order to minimize the correlation of the stimulus signal. Each visual flicker was presented on the display in accordance with the square wave signal encoded with the corresponding frequency *f* and phase φ:


$$s\left(f,\varphi ,i\right)=\frac{\mathrm{sign}\left\{\sin\left[2\pi f\left(i/240\right)+\varphi \right]\right\}+1}{2}$$

Where *sin*(⋅) generates a sine waveform, *sign*(⋅) is a sign function, and *f* and φ are the frequency and phase of the target, respectively. *i* represents the index of the frames. *s*(⋅) represents the stimulus sequence, with only two values, 0 and 1, corresponding to the lowest and highest illuminance, respectively.

#### Experimental Platform

**FIGURE 2:**
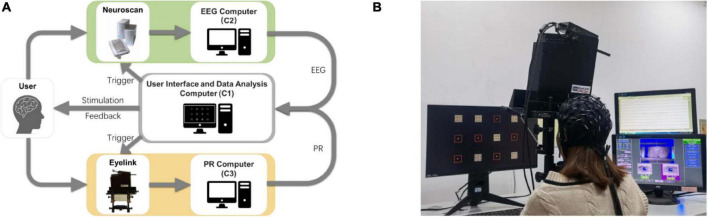
**(A)** Online experiment platform for the h-BCI based on EEG and PR. **(B)** Experimental scene of the h-BCI.

This study developed an online experiment platform for the h-BCI based on EEG and PR, including user interface and data analysis computer (C1), EEG computer (C2), and PR computer (C3), as shown in Figure 2A. The visual stimulation was presented to the subjects by C1. At the beginning of each trial, the stimulus trigger was sent from the C1’s parallel port to the EEG amplifier and the eye tracker, which was synchronized with EEG and PR signals. At the same time, the subject’s EEG and PR signals were sent from C2 and C3 to C1 in real time. Data were analyzed by C1, and auditory feedback (i.e., pronunciation of the target character in Chinese) was provided to the subject. The experimental scene diagram including electrode locations and eye tracker settings was shown in Figure 2B. In a room with normal lighting, the subjects sat comfortably on a chair 60 cm away from the stimulation monitor to complete all experiments.

### Experiment Design

**FIGURE 3:**
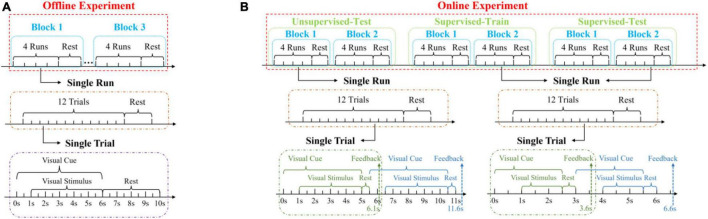
Experimental procedures of the **(A)** offline and **(B)** online experiments.

In this study, a 12-target h-BCI system based on cue-guided target selecting task was designed, which included offline and online experiments. Data collected in the offline experiment were used to optimize parameters in data analysis toward high classification accuracy. The online experiment further demonstrated and evaluated the system performance using a close-loop paradigm with online feedback. Figure 3A shows the timing procedure of the offline experiment. Each subject completed three blocks of target selections. Each block contained four runs and there was a rest time about 1 min between any two runs. Each run contained 12 trials, corresponding to 12 targets cued in a random order. Therefore, there were total 12 trials (four trials per block, three blocks) for each target in the offline experiment. The subjects were given several minutes (ranging from 5 to 10) to rest between the blocks to avoid fatigue. Each trial started with a visual cue (a red triangle), which appeared below the target stimulus for 6 s, as shown in Figure 3A. Subjects were asked to fixate at the red square in the middle of the target within 1 s and avoid eye blinking for the next 5 s when all targets started flickering together. After that, subjects can blink to reduce visual fatigue during the 4 s rest time at the end of the trial.

The online experiment included three parts: test with an unsupervised method, train and test with a supervised method, as shown in Figure 3B. These methods were mentioned in section “Classification Algorithm.” Each part contained two blocks. Different from the offline experiment, the total duration of each trial was 5.5 s (1 s sight shift, 4 s stimulus, 0.5 s rest, and 0.6 s waiting for feedback) for the unsupervised method and 3 s (1 s sight shift, 1.5 s stimulus, 0.5 s rest, and 0.6 s waiting for feedback) for the supervised method. The stimulation duration was manually selected toward optimal performance by jointly considering accuracy and ITR in offline data analysis. In addition, the visual cue of the next trial was presented after the rest time of the current trial in order to leave enough time for subjects to shift their eyesight.

In addition, a behavioral test was designed to compare the subjective perception of the 12-target stimulation interface at low (0.8–2.12 Hz) and medium (9–10.12 Hz) frequency bands. Twelve subjects were asked to fill out a questionnaire after completing 1 block of cue-guided target selecting task for each frequency band. The questionnaire (Bieger and Molina, 2010) included three parts: the comfort level (scores 1–5 correspond to very uncomfortable, uncomfortable but tolerable, somewhat uncomfortable, comfortable, very comfortable, respectively), the perception of flicker (scores 1–5 correspond to very strong flicker, strong flicker, slight flicker, perceptible flicker, imperceptible flicker, respectively) and the preference level (score 1 indicates the worst and 5 indicates the best).

### Data Analysis

#### Latency Delay

**FIGURE 4:**
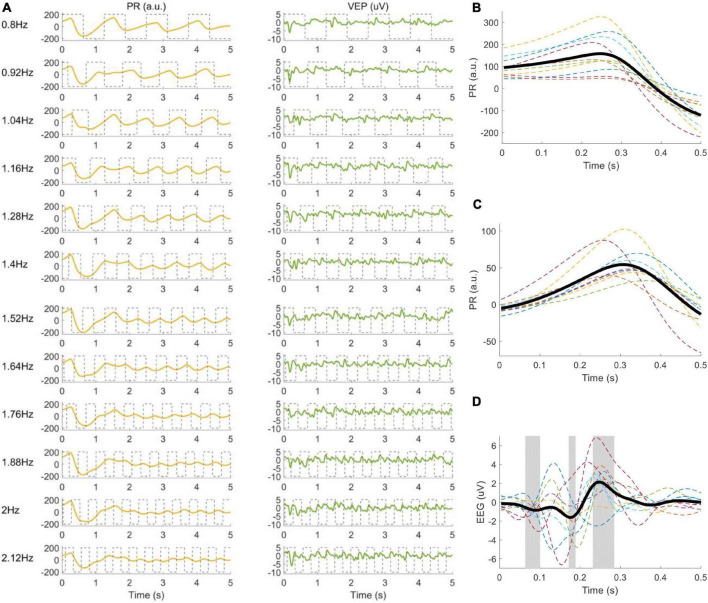
**(A)** Elicited PRs and VEPs corresponding to twelve frequencies. The gray dotted line represents the stimulus signals. **(B)** PR after stimulus onset, **(C)** PR, and **(D)** VEP when stimulus changes from 0 to 1 averaged over twelve frequencies. Each dashed line corresponds to one subject, and the thick black line is the average across all subjects. The gray areas in **(D)** represent the point-wise analysis of variance (ANOVA) results with values *p* < 0.05, to highlight the time periods with significant differences between the VEP and zeros.

The continuous EEG and PR data were divided into multiple trials according to the event channel, which recorded the stimulus onsets. The selection of time window used to extract data epochs requires an estimation of latency, which represents the time delay between stimulus onset and VEP or PR responses. The latency of VEP was set to 140 ms (Chen et al., 2015a). The latency of PR was estimated with experimental data, including the latency of the pupil constriction *lDelay*1 and the duration of the response to the stimulus onset *lDelay*2 (Muto et al., 2020). First, *lDelay*1 was observed according to the clean temporal waveforms obtained by filtering and averaging the PR signals in the offline experiment (as shown in Figure 4B). PR began to decrease at 250 ms after the stimulus onset, so *lDelay*1 was set to 250 ms. Second, *lDelay*2 was obtained by calculating the maximum classification accuracy according to the standard canonical correlation analysis (CCA) method (Lin et al., 2006), which was often used to extract the narrow-band frequency component of the signals. PR data in the offline experiment were extracted in [*lDelay*2, 5.25 s] (time 0 indicated stimulus onset) to exclude the common response after the stimulus onset and pre-processed (down-sampled to 250 Hz, band-pass filtered within 0.75∼50 Hz to reduce DC drift and power line interference). *lDelay*2 was set to different values (from 0 to 1.5 s, with an interval of 0.02 s) to calculate the corresponding CCA classification accuracy. *lDelay*2 was set to 1.2 s in this study, which corresponded to the highest accuracy, and the PR data extracted in [1.2 s, 5.25 s] were modulated by the local target’s brightness and showed steady-state periodic responses at the stimulus frequency.

#### Characteristics of Visual Evoked Potential and Pupillary Response

The temporal waveform, amplitude spectrum, and signal-to-noise ratio (SNR) were used to evaluate whether there was a frequency-locked relationship between stimulation signal and VEP or PR (Nakanishi et al., 2014). First of all, the VEP and PR signals were epoched from 0.1 s before the stimulus onset to 0.5 s after the stimulus offset, down-sampled to 250 Hz, and passed through a Chebyshev Type I band-pass filter (0.5–10 Hz). In particular, the baselines of all PR trials were corrected with respect to the mean value over the 100 ms window preceding stimulus onset. A total of 120 trials (12 trials × 10 subjects) extracted in [0 s, 5 s] (time 0 indicated stimulus onset) were averaged for each target in the offline experiment to draw clean temporal waveforms of the elicited VEP at the Oz electrode and PR of the right eye. Secondly, the data epochs in [0 s, 0.5 s] (time 0 indicated the stimulus signal changes from 0 to 1) were extracted to explore how the EEG and PR were modulated by illuminance. To remove the transient response of VEP and PR, the data in the first second after stimulus onset were excluded. Besides, the down-sampled signals filtered by a band-pass filter (0.75–50 Hz) were extracted (PR: [0.25 s, 5.25 s], VEP: [0.14 s, 5.14 s], time 0 indicated stimulus onset) for fast Fourier transform (FFT). Limited by the frequency resolution of 0.2 Hz, three frequencies (0.8, 1.4, and 2 Hz) were selected for amplitude spectrum analysis of VEP and PR. Finally, SNRs of VEP and PR were calculated as the ratio of the signal spectrum amplitude at the target to the mean of background noises:


$$\begin{array}{c} \mathrm{SNR}\left(f\right)=\\ 20\times \log_{10}\left(\frac{n\times Y\left(f\right)}{\sum_{m=1}^{n/2}\left[Y\left(f-0.2\times m\right)+Y\left(f+0.2\times m\right)\right]}\right) \end{array}$$

Where *f* denotes the stimulus frequency, *Y*(⋅)denotes the amplitude spectrum, *n* was set to 4 (i.e., 0.4 Hz on each side of the target frequency).

#### Classification Algorithm

Classification accuracy and ITR were often used to evaluate the performance of the BCI system. Firstly, the classification accuracy in the offline experiment was calculated using both supervised and unsupervised methods. Specifically, the supervised method required calibration data for training, while the unsupervised method performed classification without calibration data. For the supervised method, classification accuracy was calculated by a leave-one-out cross-validation method, which meant that 11 of the 12 runs in the offline experiment were used for training, and the remaining 1 run was used for testing in each step of validation. For the EEG, task related component analysis (TRCA) (Nakanishi et al., 2018), which can extract effective response information from EEG by maximizing the reproducibility of task-related components, has been proved highly efficient in the detection of SSVEP. EEG data extracted in [0.14 s 0.14 + *d*s] (time 0 indicated stimulus onset, *d* denoted the data length) were used for classification. *x*_*k*_, which was denoted as the EEG signals after down-sampling and band-pass filtering corresponding to the *k^th^* target, could be averaged among trials to obtain the template *X*_*k*_. The spatial filters *W* = [ω_1_, ω_2_, ⋯, ω_12_] were calculated according to the TRCA method. Then, the unlabeled EEG signal *Y* and the templates *X*_*k*_ passed through the spatial filters *W* to calculate the two-dimensional correlation coefficient *r*_*k*,1_, as:


$$r_{k,1}=\rho \left(X_{k}^{T}W,Y^{T}W\right)$$

For the PR, a template matching method was used, and the latency delay was 250 ms. Similar to EEG, the averaged two-channel PR signals *P*_*k*_ were used as the *k^th^* target’s template. Then, the correlation coefficient *r*_*k*,2_ between the unlabeled PR data *Q* and the template *P*_*k*_ was calculated as:


$$r_{k,2}=\rho \left(P_{k},Q\right)$$

For the unsupervised method, CCA was used for both VEP and PR, and the latency delay was different (VEP: 140 ms, PR: *lDelay*1 = 250 ms, *lDelay*2 = 1,200 ms). PR data were extracted in [*lDelay*2, *lDelay*1 + *d*s] (time 0 indicated stimulus onset, *d* denoted the data length).

Secondly, the filter-bank method was used to improve classification accuracy by making better use of the information on the fundamental and harmonic frequencies (Chen et al., 2015a). The EEG and PR data in the offline experiment were used to optimize the parameters of both supervised and unsupervised methods. For the supervised method, the optimization of filter banks was to generate *N* sub-bands covering multiple harmonic frequency bands *f*_1,*n*_(*n* = 1,2,⋯,*N*) with a same high cutoff frequency *f*_2_. The low cutoff frequency *f*_1,*n*_ of each sub-band was increased by a certain step size Δ*f*, as *f*_1,*n*_ = *f*_1,1_ + Δ*f*×(*n*−1) where *f*_1,1_ was set to 0.75 Hz and *f*_1,*n*_ was up to 9.75 Hz. The weight of *n^th^* sub-band was defined as *w*(*n*) = *n*^−*a*^ + *b*. Therefore, the frequency bands and weights of the sub-bands were determined by a grid search method simultaneously, where Δ*f*, *f*_2_, *a*, and *b* were limited to [0.5:0.5:9], [30:2.5:50], [0:0.25:2] and [0:0.25:1], and *N* was determined by Δ*f*. The weighted sum of correlation coefficient values corresponding to all sub-bands (i.e., $r_{k}^{1},r_{k}^{2},\cdots r_{k}^{n}$) was calculated as:


$$\tilde{r_{k}}=\sum_{n=1}^{N}w\left(n\right){\left(r_{k}^{n}\right)}^{2}$$

The correlation coefficient $\tilde{r_{k}}$ was calculated with the templates of each target, and the one with the largest correlation coefficient was determined as the target label of the test data. Besides, for the unsupervised method, the number of harmonics *N*_*h*_ of the sinusoidal signal, which was used as the reference signal in the standard CCA preprocesses (Lin et al., 2006) required to be optimized and further used in the filter-bank CCA (FBCCA) method (Chen et al., 2015a).

Thirdly, the offline experiment data were also used to calculate the weights of the hybrid data fusion method toward the highest accuracy across subjects. A decision fusion method was developed to combine the information of VEP and PR, which can further improve the accuracy of target recognition (Ma et al., 2018). The hybrid method was calculated as:


$$\begin{array}{c} R_{\mathrm{hybrid}}^{k}=\mathrm{normalize}\left(\tilde{r_{k,1}}\right)\times {\mathrm{ACC}}_{\mathrm{EEG}}^{2}+\mathrm{normalize}\left(\tilde{r_{k,2}}\right)\\ \times {\mathrm{ACC}}_{\mathrm{PR}}^{2} \end{array}$$

Where $\tilde{r_{k,1}}$ and $\tilde{r_{k,2}}$ were the correlation coefficients corresponding to EEG and PR, respectively. *normalize*(⋅) denoted the values were normalized to [0,1] interval across the 12 targets.*ACC*_*PR*_ and *ACC*_*EEG*_ were the averaged classification accuracy across all subjects. The *k^th^* character corresponding to the maximal $R_{hybrid}^{k}$ was chosen as the target character for the decision fusion method.

Finally, the optimized parameters of the filter-bank method and the weights of the fusion method based on the offline experiment were transferred to the online experiment. For the supervised method, the templates of EEG and PR and the TRCA spatial filters were re-trained in the online experiment. Besides, for both offline and online experiments, ITR (bpm) was calculated as follows:


$$\mathrm{ITR}=\left(\log_{2}M+P\log_{2}P+\left(1-P\right)\log_{2}\left(\frac{1-P}{M-1}\right)\right)\times \left(\frac{60}{T}\right)$$

Where, *M* is the total number of targets (12 in this study), *P* is the averaged classification accuracy across all the targets, and *T* is the averaged time to complete the detection of the target, including the stimulus duration and the interval time of 1.5 s.

#### Statistical Analysis

Statistical analyses were performed with SPSS software (IBM SPSS Statistics, IBM Corporation) in this study. Repeated-measures analysis of variance (RMANOVA) was used to check the difference of performance (e.g., SNR, accuracy, ITR, and so on) between different conditions (Chen et al., 2015a,^2019^; Nakanishi et al., 2018; Xu et al., 2020). Greenhouse–Geisser correction was used if the data violated the sphericity assumption by Mauchly’s test of sphericity. Bonferroni correction was applied to *post hoc* pairwise comparisons. The significance level was set to 0.05.

## Results

### Stimulus and Characteristics of Pupillary Response and Visual Evoked Potential

The waveforms of the right PRs and VEPs at the Oz electrode averaged across all subjects are illustrated in Figure 4A. PR decreased at ∼250 ms after stimulus onset (magnified in Figure 4B) for all frequencies, which was caused by the change of the overall illuminance in the visual field. Then, PR increased slightly after falling to the valley value. The clear time-locked characteristic appeared after ∼1.2 s with a latency delay of ∼300 ms (magnified in Figure 4C), which was modulated by the local target’s illuminance. For the VEP signals, the frequency and phase information of the target stimulus were accurately coded, and a pattern onset VEP (Odom et al., 2004) with negative peaks (at ∼90 ms and ∼180 ms) and a positive peak (at ∼250 ms) could be observed (in Figure 4D).

**FIGURE 5:**
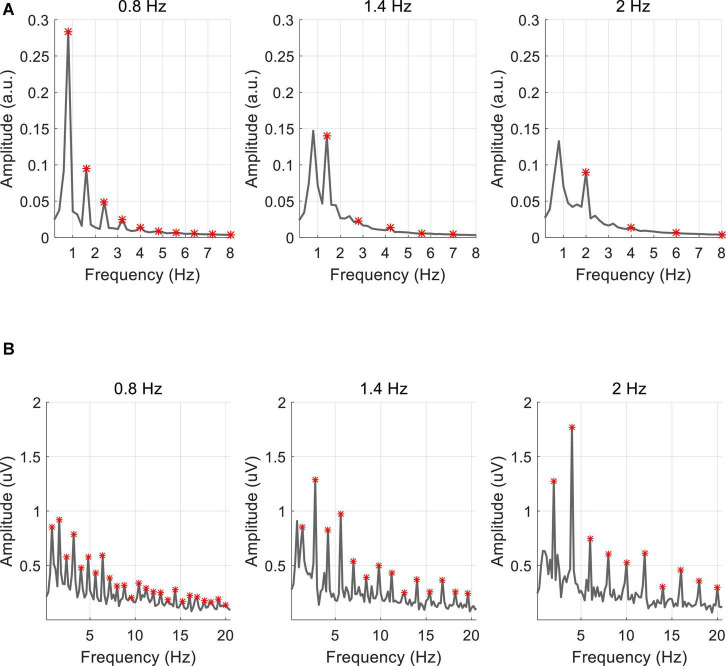
Averaged amplitude spectrum of **(A)** PR and **(B)** VEP for all subjects. The red asterisk represents the fundamental and harmonic frequencies of the stimulus frequency.

Figure 5 shows the amplitude spectra at three stimulus frequencies (0.8, 1.4, and 2 Hz) averaged across 10 subjects, marked with red asterisks at the fundamental and harmonic frequencies. As shown in Figure 5A, the amplitude spectrum of PR showed a clear response at the fundamental frequency and the amplitude decreased with increased frequencies (0.8 Hz: 0.28 a.u., 1.4 Hz: 0.14 a.u., and 2 Hz: 0.09 a.u.). The responses at the harmonic frequencies were less significant for the higher stimulus frequencies. Different from the PR, the VEP response at the Oz electrode showed significant peaks at the fundamental and harmonic frequencies (see Figure 5B). As the stimulus frequency increased, the response amplitude increased at the second harmonic (0.8 Hz: 0.92 μV, 1.4 Hz: 1.29 μV, 2 Hz: 1.77 μV).

### Comparison of the Signal-to-Noise Ratio of Pupillary Response and Visual Evoked Potential

**FIGURE 6:**
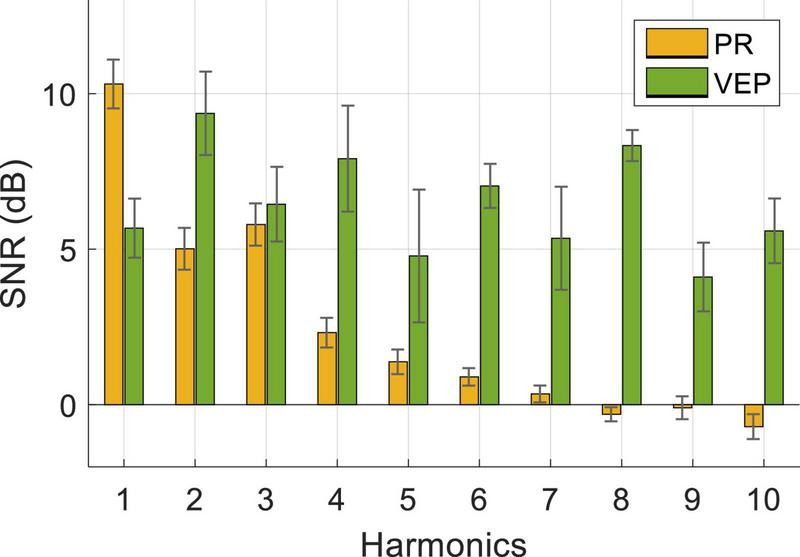
Averaged SNR of PR and VEP at Oz electrode for three stimulus frequencies and 10 subjects. The error bars represent standard errors across the subjects.

Figure 6 shows the averaged SNR of PR and VEP across three stimulus frequencies (0.8 Hz, 1.4 Hz, 2 Hz) and 10 subjects. A two-way RMANOVA shows main effects of harmonic [*F*(9,81) = 10.88, *p* < 0.05] and modality [*F*(1,9) = 38.35, *p* < 0.05], and significant interaction of these two factors [*F*(9,81) = 9.77, *p* < 0.05]. Pairwise comparisons indicate that PR had a significantly higher SNR at the fundamental frequency than harmonic frequencies (*p* < 0.05, fundamental frequency: 10.31 ± 0.79 dB, second harmonic: 5.01 ± 0.67 dB, third harmonic: 5.79 ± 0.68 dB). Differently, the SNR of VEP had no significant differences in the fundamental and harmonics frequencies (*p* > 0.05) and reached the highest at the second harmonic (9.37 ± 1.34 dB). In contrast, the SNR of PR was higher than VEP at the fundamental frequency and lower at harmonics. Pairwise comparisons revealed that the difference between PR and VEP was significant at each harmonic (*p* < 0.05), except for the third and fifth harmonics (*p* > 0.05).

### Offline Analysis

#### Supervised Method

**FIGURE 7:**
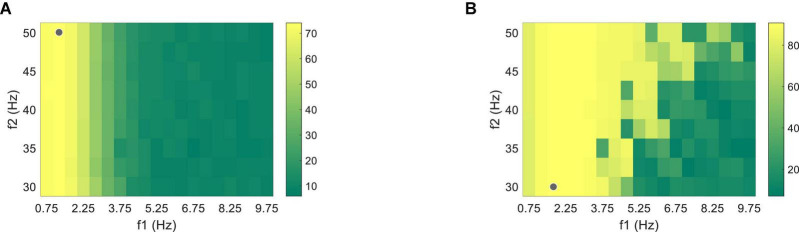
Classification accuracy of **(A)** PR and **(B)** VEP data through one band-pass filter using the supervised method at the data length of 1.5 s. The gray dots correspond to the filter settings with the highest accuracy.

**FIGURE 8:**
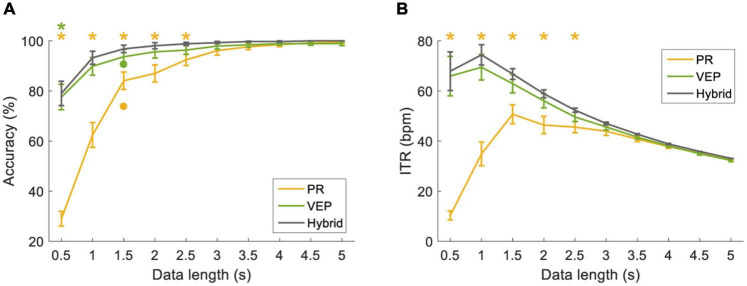
**(A)** Classification accuracy and **(B)** ITR using filter-bank supervised method at different data lengths (from 0.5 s to 5 s with a step of 0.5 s). The error bars represent standard errors across the subjects. The dot corresponds to the optimal classification accuracy using one band-pass filter. The asterisk represents a significant difference between the single-modal method and the hybrid method (pairwise comparison, *p* < 0.05).

The nine-channel EEG and binocular PR signals with the data length of 1.5 s after the visual latency were used to optimize the supervised algorithm. Firstly, Chebyshev Type I band-pass filter with a low cutoff frequency *f*_1_ (from 0.75 to 9.75 Hz, with an interval of 0.5 Hz) and a high cutoff frequency *f*_2_ (from 30 to 50 Hz, with an interval of 2.5 Hz) were adopted to calculate the classification accuracy using one band-pass filter, as shown in Figure 7. The highest classification accuracy was 74.03 ± 4.10% for PR at 1.25∼50 Hz and 90.76 ± 3.64% for VEP at 1.75∼30 Hz (the gray dots in Figure 7). The optimized results of the filter-bank method indicated that the maximal classification accuracy of PR and VEP were 84.10 ± 3.47% and 93.68 ± 2.86%, both of which were higher than the accuracy of using one band-pass filter (the asterisk in Figure 8). One-way RMANOVA indicates that the difference between the accuracy of one band-pass filter and the filter-bank method was significant [PR: *F*(1,9) = 43.75, VEP: *F*(1,9) = 8.03, *p* < 0.05]. The corresponding optimal parameters were Δ*f* = 0.5, *f*_2_ = 50, *N* = 6, *a* = 0.5, *b* = 0.5 for PR and Δ*f* = 0.5, *f*_2_ = 50, *N* = 12, *a* = 0.5, *b* = 0 for VEP.

Figure 8 compared the classification performance for the filter-bank supervised method at different data lengths (from 0.5 s to 5 s with a step of 0.5 s). The classification accuracy was the lowest at 0.5 s (PR: 28.96 ± 2.95%, VEP: 77.57 ± 5.09%, hybrid: 79.03 ± 4.85%) and increased as the data length increased. In particular, the hybrid accuracy was better than that of PR or VEP at short data length. Two-way RMANOVA shows main effects of data length [*F*(9,81) = 90.68, *p* < 0.05) and modality (*F*(2,18) = 27.51, *p* < 0.05], and there were significant interactions of data lengths and modalities [*F*(18,162) = 44.70, *p* < 0.05]. Pairwise comparisons indicate the accuracy of the hybrid method was significantly higher than that of PR with data length ≤2.5 s and was significantly higher than that of VEP with data length ≤0.5 s (*p* < 0.05). In addition, as the data length increased, ITR first increased and then decreased. At the data length of 1.5 s, the hybrid accuracy was 96.81 ± 1.49%, and the hybrid ITR was 66.77 ± 2.17 bpm (PR: 50.72 ± 3.79 bpm, VEP: 62.91 ± 3.69 bpm). Two-way RMANOVA reveals main effects of data length [*F*(9,81) = 27.68, *p* < 0.05] and modality [*F*(2,18) = 29.74, *p* < 0.05], and significant interactions [*F*(18,162) = 39.09, *p* < 0.05]. Pairwise comparisons indicate that the difference between the hybrid method and PR was significant at data length ≤2.5 s (*p* < 0.05).

**FIGURE 9:**
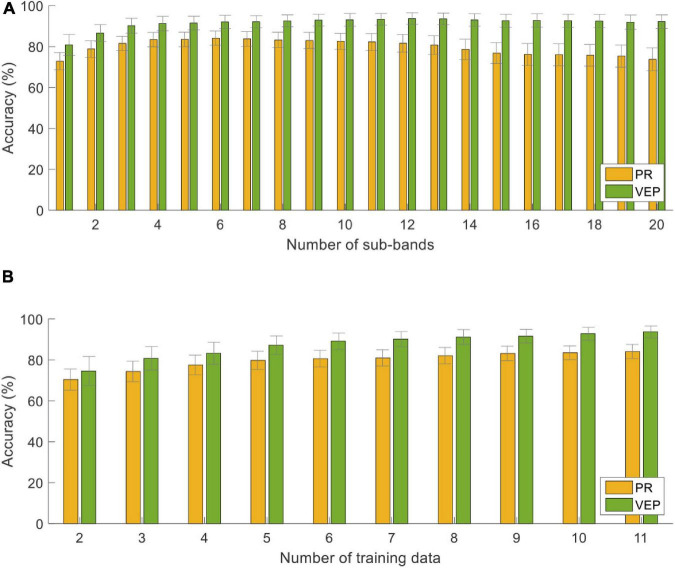
Classification accuracy with different **(A)** numbers of sub-bands and **(B)** numbers of training trials using the supervised method at the data length of 1.5 s. The error bars represent standard errors across the subjects.

Figure 9 shows the classification accuracy using different parameters: the number of sub-bands (*N* from 1 to 20) and the number of training trials (*N*_*t*_ from 1 to 11) at the data length of 1.5 s. Overall, the accuracy of PR was always lower than that of VEP. As the *N* value increased, the accuracy of PR increased first and reached the maximum at *N* = 6, then decreased and reached the lowest at *N* = 20 (73.75 ± 5.62%). It suggests that only the first several harmonics of PR contribute to the classification. For VEP, the accuracy was the lowest at *N* = 1 (80.83 ± 5.12%), then increased as the number of sub-bands increased and tended to be saturated. In addition, one-way RMANOVA reveals that the accuracy of PR or VEP has a significant difference between different numbers of training data [PR: *F*(9,81) = 27.08, VEP: *F*(9,81) = 14.40, *p* < 0.05]. Pairwise comparisons show that the accuracy will not increase significantly when *N*_*t*_ is greater than 8 (*p* > 0.05, PR: 82.08 ± 3.97%, VEP: 91.18 ± 3.61% at *N*_*t*_ = 8). Therefore, this study set the number of training trials *N*_*t*_ = 8 to reduce the training cost in the online experiment.

#### Unsupervised Method

**FIGURE 10:**
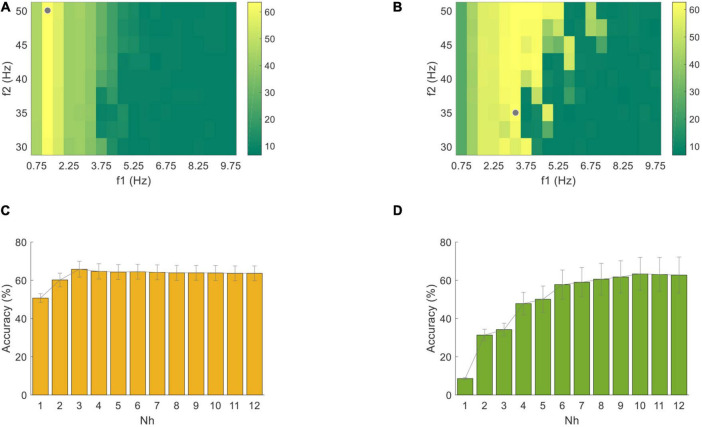
Classification accuracy of **(A)** PR and **(B)** VEP data through one band-pass filter using the standard CCA method with a data length of 4 s. The gray dots correspond to the filter settings with the highest accuracy. Classification accuracy of **(C)** PR and **(D)** VEP with different numbers of harmonics in the standard CCA method. The error bars represent standard errors across the subjects.

**FIGURE 11:**
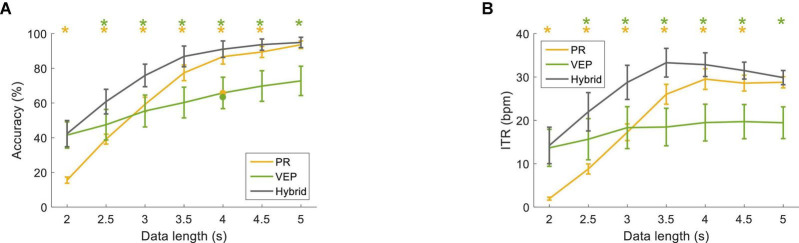
**(A)** Classification accuracy and **(B)** ITR using the FBCCA method at different data lengths (from 2 s to 5 s with a step of 0.5 s). The error bars represent standard errors across the subjects. The dot corresponds to the optimal classification accuracy using one band-pass filter. The asterisk represents a significant difference between the single-modal method and the hybrid method (pairwise comparison, *p* < 0.05).

For the unsupervised algorithm, the optimization was based on the VEP and PR data with a length of 4 s starting from the visual latency. First, the classification accuracies of the standard CCA method corresponding to different band-pass filters at *N*_*h*_ = 12 were shown in Figures 10A,B. The highest classification accuracy was 63.61 ± 3.84% for PR at 1.25∼50 Hz and 62.71 ± 9.42% for VEP at 3.25∼35 Hz (the gray dots in Figures 10A,B). Then, the classification accuracies using different *N*_*h*_ values (from 1 to 12) in this optimal band-pass filter were compared, as shown in Figures 10C,D. As the number of *N*_*h*_ increased, the classification accuracy of PR increased first and then decreased, and the highest was 65.76 ± 4.13% when *N*_*h*_ = 3. The accuracy of VEP increased when *N*_*h*_ increased and reached the highest when *N*_*h*_ = 10 (63.33 ± 8.64%). Therefore, this study used *N*_*h*_ = 3 for PR and *N*_*h*_ = 10 for VEP in all standard CCA preprocesses for the FBCCA method. The optimized filter-bank results indicated that the maximal classification accuracy of PR and VEP were 86.74 ± 4.28% and 65.83 ± 9.07%, respectively. The corresponding optimal parameters were Δ*f* = 0.5, *f*_2_ = 50, *N* = 19, *a* = 0.25, *b* = 1 for PR and Δ*f* = 0.5, *f*_2_ = 50, *N* = 10, *a* = 0, *b* = 0 for VEP. Compared with the standard CCA method (the asterisk in Figure 11), the filter bank related improvement of PR was greater than that of VEP. One-way RMANOVA indicates that the improvement of PR was significant [*F*(1,9) = 68.89, *p* < 0.05], while that of VEP was not significant [*F*(1,9) = 4.38, *p* > 0.05].

Figure 11 shows the classification performance with the optimized parameters for the FBCCA method at different data lengths (from 2 s to 5 s with a step of 0.5 s). The accuracy of PR was lower than VEP at the data length of 2 s (hybrid: 42.36 ± 7.54%, PR: 15.56 ± 1.77%, VEP: 41.53 ± 7.54%). The accuracy increased with the increase of data length, and the accuracy of PR was higher than that of VEP with data lengths ≥3 s. The classification accuracy of the hybrid method was higher than that of PR and VEP and reached 91.04 ± 4.76% at 4 s. Two-way RMANOVA shows main effects of data length [*F*(6,54) = 164.77, *p* < 0.05] and modality [*F*(2,18) = 5.72, *p* < 0.05], and significant interactions of the two factors [*F*(12,108) = 19.39, *p* < 0.05]. Moreover, ITR increased as the data length increased and reached 32.85 ± 2.74 bpm at 4 s for the hybrid method (PR: 29.52 ± 2.38 bpm, VEP: 19.49 ± 4.25 bpm). Two-way RMANOVA shows main effects of data length [*F*(6,54) = 49.07, *p* < 0.05] and modality [*F*(2,18) = 5.92, *p* < 0.05], and there were significant interactions [*F*(12,108) = 16.35, *p* < 0.05]. Pairwise comparisons indicate that the accuracy and ITR of the hybrid method were significantly higher than that of PR with data length ≤4.5 s and were significantly higher than that of VEP with data length ≥2.5 s (*p* < 0.05).

**FIGURE 12:**
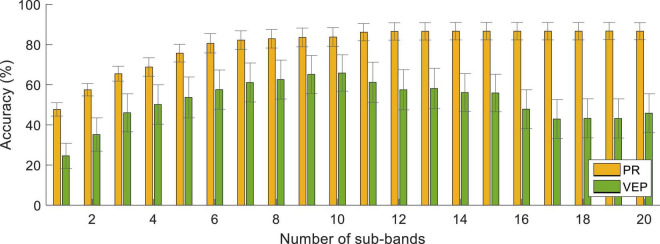
Classification accuracy with different numbers of sub-bands using the unsupervised method at the data length of 4 s. The error bars represent standard errors across the subjects.

Figure 12 shows the classification accuracy of the FBCCA method with different numbers of sub-bands (*N* from 1 to 15) at the data length of 4 s. For PR, the classification accuracy was the lowest at *N* = 1 (47.71 ± 3.33%), then increased slightly as the number of sub-bands increased. The accuracy of VEP increased first and then decreased after reaching the maximum at *N* = 10.

### Online Hybrid BCI Performance

Table 1 lists the results of the online cue-guided target selecting task using the supervised method. The averaged hybrid accuracy across 10 subjects was 94.90 ± 2.34% (PR: 79.90 ± 4.11%, VEP: 89.17 ± 4.20%), and the corresponding ITR was 64.35 ± 3.07 bpm (PR: 45.92 ± 4.23 bpm, VEP: 57.55 ± 5.04 bpm). Table 2 lists the results of the online experiment using the unsupervised method. The averaged hybrid accuracy across 10 subjects was 91.88 ± 3.68% (PR: 84.06 ± 4.52%, VEP: 61.56 ± 7.93%), and the corresponding ITR was 33.19 ± 2.38 bpm (PR: 27.91 ± 2.66 bpm, VEP: 16.90 ± 4.00 bpm). One-way RMANOVA indicates that the differences between the offline and online experiment were not significant (for the supervised method, the accuracy of PR: *F*(1,9) = 3.44, the accuracy of VEP: *F*(1,9) = 3.17, the accuracy of hybrid: *F*(1,9) = 3.31, the ITR of PR: *F*(1,9) = 4.40, the ITR of VEP: *F*(1,9) = 3.49, the ITR of hybrid: *F*(1,9) = 3.09; for the unsupervised method, the accuracy of PR: *F*(1,9) = 1.21, the accuracy of VEP: *F*(1,9) = 1.18, the accuracy of hybrid: *F*(1,9) = 0.22, the ITR of PR: *F*(1,9) = 0.97, the ITR of VEP: *F*(1,9) = 1.70, the ITR of hybrid: *F*(1,9) = 0.11; *p* > 0.05 for all conditions).

**TABLE 1:** Results of online cued-guided experiment (supervised method).

Subject	Accuracy (%)			ITR (bpm)		
	PR	VEP	Hybrid	PR	VEP	Hybrid
Sub1	76.04	100.00	100.00	41.21	71.70	71.70
Sub2	94.79	97.92	100.00	62.92	68.19	71.70
Sub3	90.63	96.88	98.96	57.42	66.88	69.94
Sub4	89.58	77.08	91.67	55.92	41.00	58.55
Sub5	55.21	67.71	76.04	21.80	32.63	40.78
Sub6	66.67	95.83	94.79	31.48	64.68	63.37
Sub7	88.54	66.67	91.67	54.17	31.52	58.99
Sub8	87.50	98.96	100.00	53.04	69.94	71.70
Sub9	67.71	96.88	98.96	33.57	66.88	69.94
Sub10	82.29	93.75	96.88	47.62	62.06	66.88
Mean ± ste	79.90 ± 4.11	89.17 ± 4.20	94.90 ± 2.34	45.92 ± 4.23	57.55 ± 5.04	64.35 ± 3.07

**TABLE 2:** Results of online cued-guided experiment (unsupervised method).

Subject	Accuracy (%)			ITR (bpm)		
	PR	VEP	Hybrid	PR	VEP	Hybrid
Sub1	69.79	98.96	97.92	18.95	38.15	37.19
Sub2	93.75	73.96	100.00	33.61	21.18	39.11
Sub3	96.88	54.17	98.96	36.24	11.55	38.15
Sub4	84.38	33.33	83.33	27.20	4.54	26.88
Sub5	52.08	33.33	62.50	10.62	5.09	15.09
Sub6	88.54	71.88	97.92	29.89	19.42	37.19
Sub7	96.88	41.67	95.83	36.24	6.97	35.28
Sub8	84.38	97.92	97.92	27.65	37.19	37.44
Sub9	95.83	71.88	96.88	35.52	19.34	36.48
Sub10	78.13	38.54	87.50	23.15	5.53	29.11
Mean ± ste	84.06 ± 4.52	61.56 ± 7.93	91.88 ± 3.68	27.91 ± 2.66	16.90 ± 4.00	33.19 ± 2.38

### Individual Differences

**FIGURE 13:**
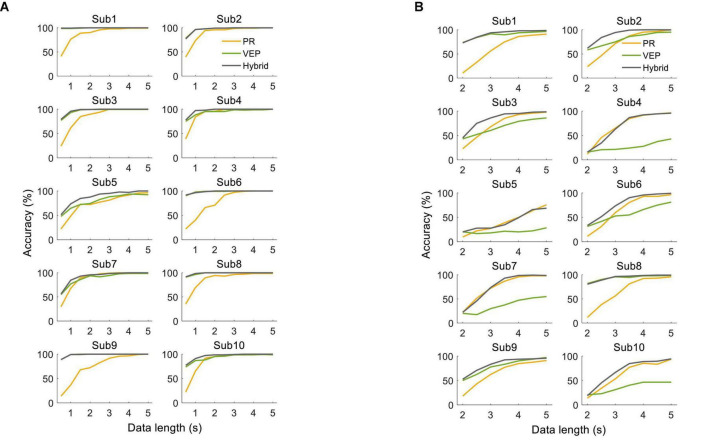
Classification accuracy using **(A)** supervised method and **(B)** unsupervised method for each subject in the offline experiment.

There were clear individual differences in the system performance. Figure 13 shows the classification accuracy for each subject in the offline experiment. For the supervised method, some subjects had significantly higher accuracy of VEP than PR with short data lengths. The hybrid accuracy was higher than that of VEP for Sub4, Sub5, Sub7, and Sub10. For the unsupervised method, significant differences appeared among the subjects. For some subjects, EEG accuracy was always higher than that of PR (Sub1, Sub8, and Sub9), while PR accuracy was always higher than that of EEG in other subjects (Sub4, Sub5, Sub7, and Sub10). Besides, several subjects had higher accuracy of PR than EEG only when the data length was long (Sub2, Sub3, and Sub6). Notably, the hybrid accuracy was higher than PR and VEP for most subjects, especially when the data length was short. Similar conclusions can be obtained from the results of the online experiment, as shown in Tables 1, 2. For the supervised method, Sub4 and Sub7 had a higher accuracy of PR than EEG, while the other 8 subjects showed the opposite condition. However, the hybrid classification performance was higher than PR and EEG for all subjects, except for Sub6. In particular, the accuracy of VEP was 66.67%, and the hybrid accuracy reached 91.67% after combining PR characteristics, with an increase of 25.00% for Sub7. For the unsupervised method, two subjects (Sub1 and Sub8) had a lower accuracy of PR than VEP. It can also be observed that the hybrid classification performance has been improved for all subjects compared to VEP, except for Sub1. Especially for Sub7, the hybrid accuracy reached 95.83%, which was 54.16% higher than that of VEP. These results indicate that the proposed hybrid BCI yielded a considerable benefit for some subjects and thereby alleviated the problem of BCI illiteracy.

### Behavioral Test

One-way RMANOVA shows that the behavioral scores of the low-frequency band were significantly higher than that of the medium-frequency band (low vs. medium, comfort level: 3.42 ± 0.31 vs. 1.75 ± 0.22, *F*(1,11) = 34.38, *p* < 0.05; perception of flicker: 2.50 ± 0.23 vs. 1.75 ± 0.18, *F*(1,11) = 9.00, *p* < 0.05; preference level: 3.17 ± 0.34 vs. 1.92 ± 0.23, *F*(1,11) = 14.47, *p* < 0.05), which meant that the low-frequency stimulus used in the proposed h-BCI was a more comfortable and favorable stimulus than the alpha frequency band used in the traditional SSVEP-BCI.

## Discussion

Compared with other traditional SSVEP-BCI in the alpha frequency range, the h-BCI system based on the low-frequency stimulations developed in this study is more comfortable and applicable for all users. This study performed a pre-experiment to compare the subjective perception of visual stimuli (Chien et al., 2017) with different frequencies (from 1 to 60 Hz, with an interval of 1 Hz). The averaged results with 12 subjects showed that the subjects’ comfortableness and preference level at the low-frequency (e.g., 1 Hz) were better than the alpha frequency range (e.g., 10 Hz). Compared with the high-speed SSVEP-BCIs (Chen et al., 2015a,b; Nakanishi et al., 2018), the relatively low classification accuracy and ITR in the proposed system can be explained in several aspects. First of all, the poor performance of VEP for the unsupervised method may be caused by the low response amplitude at the low-frequency band. Herrmann compared the SSVEP response evoked by the flicker light at frequencies from 1 Hz to 100 Hz (in 1 Hz step) and found that the amplitude reached the highest in the medium frequency band (maximum at ∼15 Hz) (Herrmann, 2001). The response amplitude and SNR decreased significantly below 10 Hz (Wang et al., 2005), which was related to the higher level of the spontaneous EEG oscillations (Gao et al., 2003). Therefore, the frequency detection techniques used in the unsupervised method were affected by the background noise, while the TRCA-based spatial filtering techniques used in the supervised method were effective to remove background EEG and enhance SNR (Nakanishi et al., 2018). Second, the narrow frequency range and separable frequency interval of PR limit the number of targets and affect ITR. For the frequency range, Naber verified that the PR only responded to repetitive onsets and offsets of stimuli at 0.3–2.3 Hz, not at 3.4 Hz (Naber et al., 2013). Regarding to the frequency interval, Muto classified two visual stimulus targets with a luminance-modulation frequency between 0.75 and 2.5 Hz and found that the classification accuracy with the frequency interval of 0.06 Hz was poor (Muto et al., 2020). Third, data length also influences ITR. Compared with the alpha frequency band, which was commonly used in the traditional SSVEP-BCI, the low-frequency band took more time to complete the same number of stimulus periods to obtain sufficient effective information.

Compared with other SSVEP-BCI systems with better user experience than the system using stimulations in the alpha frequency range, the proposed h-BCI system offers higher BCI performance. Chang generated an amplitude-modulated (AM) stimulus (Chang et al., 2014) as the product of two sine waves, including a carrier frequency higher than 40 Hz to reduce eye fatigue and a modulating frequency ranged around the alpha band (9–12 Hz) to utilize harmonic information. Online experiments showed that the average accuracy of the six-target classification task was 91.2%, resulting in an ITR of 30 bpm. Dreyer adapted a frequency-modulated (FM) stimulation from the auditory domain (Dreyer and Herrmann, 2015), with a modulation frequency of 10 Hz and a carrier frequency ranging from 20 to 100 Hz. The subjective flicker perception of FM-SSVEPs with carrier frequencies above 30 Hz was slight or even imperceptible, while the amplitude of FM-SSVEPs (e.g., carrier frequency = 30 Hz: 0.36 μV) remained the same as that of SSVEPs evoked rectangularly (0.44 μV) or sinusoidally (0.42 μV). BCI performances between FM and sinusoidal (SIN) SSVEPs were compared using a four-target classification paradigm (Dreyer et al., 2017). The highest classification accuracy was 86% for FM (11 s epochs) and 95% for SIN (11 s epochs), and the highest ITR was 13.91 bpm for FM (2 s epochs) and 24.21 bpm for SIN (1 s epochs). Jiang developed a four-class phase-coded SSVEP-BCI by imperceptible flickers at 60 Hz (Jiang et al., 2019). The system achieved a classification accuracy of 92.71 ± 7.56% in the online experiment and an ITR of 18.81 ± 4.74 bpm.

This study proved that the combination of PR and VEP can facilitate the implementation of visual BCIs with low-frequency stimulations, and the proposed system achieved high classification accuracy and short stimulus duration compared with the other existing h-BCIs based on PR and VEP. There was an improvement for the hybrid method compared to PR and VEP for some subjects, especially when the data length was short, and the accuracy was not saturated. However, there are ways to improve the system performance and practicability of the proposed h-BCI. Firstly, other unsupervised algorithms can be explored to improve the SNR at the low-frequency band with low response amplitude and facilitate the classification of VEPs. The hybrid accuracy can be improved by customizing the weighted coefficient of the hybrid method for each participant due to the clear individual differences in the system performance. Secondly, the optimization of the grid stimulus paradigm may be helpful for increasing ITR and reducing the flickering sensation of visual stimulation, including the spatial frequency, the proportion of stimulation area, and illuminance (Ming et al., 2021). More entrained VEPs can be generated and enhanced by stimuli containing spatial contrast than the spatially uniform stimuli (Williams et al., 2004), and the spatial frequency can be optimized to maximize the ITR and reduce the visual irritation (Waytowich et al., 2017). The proportion of stimulation area and stimulation illuminance can be minished to reduce the perception of flicker and maintain the system performance. Finally, in order to be convenient to use, the system should be compact and portable (Gao et al., 2003). This study was completed in the laboratory using high-precision eye tracker and EEG amplifier. A wearable hybrid BCI system that can be used in daily life may be realized with a high-precision consumer-grade eye tracker and a wearable EEG device in the future. By addressing these issues, the h-BCI based on VEP and PR can be further improved and be potential for more practical applications.

## Conclusion

This study designed a 12-target h-BCI system with low stimulation frequencies of 0.8–2.12 Hz, which can simultaneously induce significant PR and VEP responses. The SNR of PR was higher than VEP at the fundamental frequency but lower than VEP at harmonics. PR and VEP data were recorded in the offline experiments to optimize the algorithm parameters, and the system performance was verified through online experiments. The averaged accuracy across 10 subjects was 94.90 ± 2.34% at the data length of 1.5 s for the supervised method and 91.88 ± 3.68% at 4 s for the unsupervised method, corresponding to the ITR of 64.35 ± 3.07 bpm and 33.19 ± 2.38 bpm in the online experiment. The h-BCI performance was better than PR or VEP for some subjects, especially for the short data length and unsaturated accuracy. The proposed h-BCI provides a good solution to a practical BCI with balanced system performance and user experience.

## Data Availability Statement

The raw data supporting the conclusions of this article will be made available by the authors, without undue reservation.

## Ethics Statement

The studies involving human participants were reviewed and approved by the Research Ethics Committee of Tsinghua University. The patients/participants provided their written informed consent to participate in this study.

## Author Contributions

LJ developed the experimental system, performed data collection and data analysis, and wrote the manuscript. XL developed the experimental system and performed data analysis. WP and XG revised the manuscript. YW supervised the study. All authors contributed to the article and approved the submitted version.

## Conflict of Interest

The authors declare that the research was conducted in the absence of any commercial or financial relationships that could be construed as a potential conflict of interest.

## Publisher’s Note

All claims expressed in this article are solely those of the authors and do not necessarily represent those of their affiliated organizations, or those of the publisher, the editors and the reviewers. Any product that may be evaluated in this article, or claim that may be made by its manufacturer, is not guaranteed or endorsed by the publisher.

## References

[B1] Allison B. Luth T. Valbuena D. Teymourian A. Volosyak I. Graser A. (2010). BCI demographics: how many (and What Kinds of) people can use an SSVEP BCI? *IEEE Trans. Neural Systems Rehabilitation Eng.* 18 107–116. 10.1109/TNSRE.2009.2039495 20083463

[B2] Bieger J. Molina G. G. (2010). *Light Stimulation Properties to Influence Brain Activity: A Brain-CoMputer Interface application.* Amsterdam: Philips Research.

[B3] Bouma H. (1962). Size of the static pupil as a function of wave-length and luminosity of the light incident on the human eye. *Nature* 193 690–691. 10.1038/193690a0 13871842

[B4] Brainard D. (1997). The psychophysics toolbox. *Spatial Vision* 10 433–436. 9176952

[B5] Brunner C. Allison B. Z. Krusienski D. J. Kaiser V. Gr M. P. Pfurtscheller G. (2010). Improved signal processing approaches in an offline simulation of a hybrid brain–computer interface. *J. Neurosci. Methods* 188 165–173. 10.1016/j.jneumeth.2010.02.002 20153371 PMC3422070

[B6] Chang M. H. Baek H. J. Lee S. M. Park K. S. (2014). An amplitude-modulated visual stimulation for reducing eye fatigue in SSVEP-based brain–computer interfaces. *Clin. Neurophysiol.* 125 1380–1391. 10.1016/j.clinph.2013.11.016 24368034

[B7] Chen J. Maye A. Engel A. K. Wang Y. Zhang D. (2019). Simultaneous decoding of eccentricity and direction information for a single-flicker SSVEP BCI. *Electronics* 8:1554. 10.3390/electronics8121554

[B8] Chen X. Wang Y. Gao S. Jung T. P. Gao X. (2015a). Filter bank canonical correlation analysis for implementing a high-speed SSVEP-based brain-computer interface. *J. Neural Eng.* 12:046008. 10.1088/1741-2560/12/4/046008 26035476

[B9] Chen X. Wang Y. Nakanishi M. Gao X. Jung T. P. Gao S. (2015b). High-speed spelling with a noninvasive brain-computer interface. *Proc. Natl. Acad. Sci. U S A.* 112 E6058–E6067. 10.1073/pnas.1508080112 26483479 PMC4640776

[B10] Chien Y. Y. Lin F. C. Zao J. K. Chou C. C. Huang Y. P. Kuo H. Y. (2017). Polychromatic SSVEP stimuli with subtle flickering adapted to brain-display interactions. *J. Neural Eng.* 14:016018. 10.1088/1741-2552/aa550d 28000607

[B11] Crawford B. H. (1936). The dependence of pupil size upon external light stimulus under static and variable conditions. *Proc. R. Soc. B* 121 376–395. 10.1098/rspb.1936.0072

[B12] De’Sperati C. Roatta S. Baroni T. (2020). Decoding overt shifts of attention in depth through pupillary and cortical frequency tagging. *J. Neural Eng.* 10.1088/1741-2552/ab8e8f Online ahead of print. 32348980

[B13] Dreyer A. M. Herrmann C. S. (2015). Frequency-modulated steady-state visual evoked potentials: a new stimulation method for brain–computer interfaces. *J. Neurosci. Methods* 241 1–9. 10.1016/j.jneumeth.2014.12.004 25522824

[B14] Dreyer A. M. Herrmann C. S. Rieger J. W. (2017). Tradeoff between user experience and BCI classification accuracy with frequency modulated steady-state visual evoked potentials. *Front. Hum. Neurosci.* 11:391. 10.3389/fnhum.2017.00391 28798676 PMC5526890

[B15] Fisher R. S. Harding G. Erba G. Barkley G. L. Wilkins A. (2005). Photic- and pattern-induced seizures: a review for the Epilepsy Foundation of America Working Group. *Epilepsia* 46 1426–1441. 10.1111/j.1528-1167.2005.31405.x 16146439

[B16] Floriano A. Carmona V. L. Diez P. F. Bastos-Filho T. F. (2019). A study of SSVEP from below-the-hairline areas in low-, medium-, and high-frequency ranges. *Res. Biomed. Eng.* 35 71–76. 10.1007/s42600-019-00005-2

[B17] Gandhi V. (2007). *Toward Brain-computer Interfacing.* Cambridge, MA: MIT Press. 10.1111/j.1468-1331.2008.02463.x

[B18] Gao S. Wang Y. Gao X. Hong B. (2014). Visual and auditory brain-computer interfaces. *IEEE Trans. Biomed. Eng.* 61 1436–1447. 10.1109/TBME.2014.2300164 24759277

[B19] Gao X. Xu D. Cheng M. Gao S. (2003). A BCI-based environmental controller for the motion-disabled. *IEEE Trans. Neural Systems Rehabilitation Eng.* 11 137–140. 10.1109/TNSRE.2003.814449 12899256

[B20] Harding G. F. Harding P. F. (2010). Televised material and photosensitive epilepsy. *Epilepsia* 40 65–69. 10.1111/j.1528-1157.1999.tb00909.x 10487176

[B21] Hartmann E. Lachenmayr B. Brettel H. (1978). The peripheral critical flicker frequency. *Vision Res.* 19 1019–1023. 10.1016/0042-6989(79)90227-X 532115

[B22] Herrmann C. S. (2001). Human EEG responses to 1–100 Hz flicker: resonance phenomena in visual cortex and their potential correlation to cognitive phenomena. *Exp. Brain Res.* 137 346–353. 10.1007/s002210100682 11355381

[B23] Hoffmann U. Fimbel E. J. Keller T. (2009). “Brain-computer interface based on high frequency steady-state visual evoked potentials: a feasibility study,” in *Proceedings of the 2009 4th International IEEE/EMBS Conference on Neural Engineering*, (Piscataway, NJ: IEEE).

[B24] Hoffmann U. Vesin J. M. Ebrahimi T. Diserens K. (2008). An efficient P300-based brain-computer interface for disabled subjects. *J. Neurosci. Methods* 167 115–125. 10.1016/j.jneumeth.2007.03.005 17445904

[B25] Hong K. S. Jawad K. M. (2017). Hybrid brain–computer interface techniques for improved classification accuracy and increased number of commands: a review. *Front. Neurorobotics* 11:35. 10.3389/fnbot.2017.00035 28790910 PMC5522881

[B26] Jiang L. Li X. Wang Y. Pei W. Chen H. (2020). “Comparison of pupil size and visual evoked potentials under 1-6Hz visual stimulation,” in *Proceedings of the 42nd Annual International Conference of the IEEE Engineering in Medicine and Biology Society (EMBC)*, (Piscataway, NJ: IEEE), 10.1109/embc44109.2020.9175893 33018649

[B27] Jiang L. Wang Y. Pei W. Chen H. (2019). “A four-class phase-coded SSVEP BCI at 60Hz using refresh rate,” in *Proceedings of the 41st Annual International Conference of the IEEE Engineering in Medicine and Biology Society (EMBC)*, (Piscataway, NJ: IEEE). 10.1109/EMBC.2019.8857326 31947290

[B28] Kret M. E. Sjak-Shie E. E. (2019). Preprocessing pupil size data: guidelines and code. *Behav. Res. Methods* 51 1336–1342. 10.3758/s13428-018-1075-y 29992408 PMC6538573

[B29] Kuebier A. Furdea A. Halder S. Hammer E. M. Nijboer F. Kotchoubey B. (2009). A brain-computer interface controlled auditory event-related potential (p300) spelling system for locked-in patients. *Ann. N. Y. Acad. Sci.* 1157 90–100. 10.1111/j.1749-6632.2008.04122.x 19351359

[B30] Lin Z. Zhang C. Wu W. Gao X. (2006). Frequency recognition based on canonical correlation analysis for SSVEP-Based BCIs. *IEEE Trans. Biomed. Eng.* 53 2610–2614. 10.1109/TBME.2006.889197 17152442

[B31] Luo A. Sullivan T. J. (2010). A user-friendly SSVEP-based brain–computer interface using a time-domain classifier. *J. Neural Eng.* 7:026010. 10.1088/1741-2560/7/2/026010 20332551

[B32] Ma X. Yao Z. Wang Y. Pei W. Chen H. (2018). “Combining brain-computer interface and eye tracking for high-speed text entry in virtual reality,” in *Proceedings of the 23rd International Conference on Intelligent User Interfaces*, (New York, NY: ACM), 263–267. 10.1145/3172944.3172988

[B33] Mao X. Li W. Hu H. Jin J. Chen G. (2020). Improve the classification efficiency of high-frequency phase-tagged SSVEP by a recursive bayesian-based approach. *IEEE Trans. Neural. Syst. Rehabil. Eng.* 28 561–572. 10.1109/TNSRE.2020.2968579 31985429

[B34] Mason S. G. Bashashati A. Fatourechi M. Navarrog K. F. (2007). A comprehensive survey of brain interface technology designs. *Ann. Biomed. Eng.* 35 137–169. 10.1007/s10439-006-9170-0 17115262

[B35] Ming G. Pei W. Chen H. Gao X. Wang Y. (2021). Optimizing spatial properties of a new checkerboard-like visual stimulus for user-friendly SSVEP-based BCIs. *J. Neural Eng.* 18:056046. 10.1088/1741-2552/ac284a 34544060

[B36] Muto Y. Miyoshi H. Kaneko H. (2020). Eye-gaze information input based on pupillary response to visual stimulus with luminance modulation. *PLoS One* 15:e0226991. 10.1371/journal.pone.0226991 31917794 PMC6952090

[B37] Naber M. Alvarez G. Nakayama K. (2013). Tracking the allocation of attention using human pupillary oscillations. *Front. Psychol.* 4:919. 10.3389/fpsyg.2013.00919 24368904 PMC3857913

[B38] Nakanishi M. Wang Y. Chen X. Wang Y. T. Gao X. Jung T. P. (2018). Enhancing detection of SSVEPs for a high-speed brain speller using task-related component analysis. *IEEE Trans. Biomed. Eng.* 65 104–112. 10.1109/TBME.2017.2694818 28436836 PMC5783827

[B39] Nakanishi M. Wang Y. Wang Y. T. Mitsukura Y. Jung T. P. (2014). A high-speed brain speller using steady-state visual evoked potentials. *Int. J. Neural Syst.* 24:1450019. 10.1142/S0129065714500191 25081427

[B40] Odom J. V. Bach M. Barber C. Brigell M. Marmor M. F. Tormene A. P. (2004). Visual evoked potentials standard (2004). *Doc. Ophthalmol.* 108 115–123. 10.1023/B:DOOP.0000036790.67234.22 15455794

[B41] Ponzio F. Villalobos A. Mesin L. De’sperati C. Roatta S. (2019). A human-computer interface based on the “voluntary” pupil accommodative response. *Int. J. Hum. Comp. Stud.* 126 53–63. 10.1016/j.ijhcs.2019.02.002

[B42] Sebastiaan M. Jean-Baptiste M. Lotje V. Stefan V. Hedderik V. R. (2016). The mind-writing pupil: a human-computer interface based on decoding of covert attention through pupillometry. *PLoS One* 11:e0148805. 10.1371/journal.pone.0148805 26848745 PMC4743834

[B43] Vidal J. J. (1977). Real-time detection of brain events in EEG. *Proc. IEEE* 65 633–641. 10.1109/PROC.1977.10542

[B44] Vidaurre C. Blankertz B. (2009). Towards a cure for BCI illiteracy. *Brain Topogr.* 23 194–198. 10.1007/s10548-009-0121-6 19946737 PMC2874052

[B45] Volosyak I. (2011). SSVEP-based Bremen-BCI interface–boosting information transfer rates. *J. Neural Eng.* 8:036020. 10.1088/1741-2560/8/3/036020 21555847

[B46] Volosyak I. Gembler F. Stawicki P. (2017). Age-related differences in SSVEP-based BCI performance. *Neurocomputing* 250 57–64. 10.1016/j.neucom.2016.08.121

[B47] Wang Y. Gao X. Hong B. Jia C. Gao S. (2008). Brain-Computer interfaces based on visual evoked potentials. *IEEE Eng. Med. Biol. Mag.* 27 64–71. 10.1109/MEMB.2008.923958 18799392

[B48] Wang Y. Wang R. Gao X. Gao S. (2005). “Brain-computer interface based on the high-frequency steady-state visual evoked potential,” in *Proceedings of the 1st International Conference on Neural Interface & Control*, (Piscataway, NJ: IEEE).

[B49] Waytowich N. R. Yamani Y. Krusienski D. J. (2017). Optimization of checkerboard spatial frequencies for steady-state visual evoked potential brain–computer interfaces. *IEEE Trans. Neural. Syst. Rehabil. Eng.* 25 557–565. 10.1109/TNSRE.2016.2601013 27542113

[B50] Williams P. E. Mechler F. Gordon J. Shapley R. Hawken M. J. (2004). Entrainment to video displays in primary visual cortex of macaque and humans. *J. Neurosci.* 24:8278. 10.1523/JNEUROSCI.2716-04.2004 15385611 PMC6729686

[B51] Woodhouse J. M. (1975). The effect of pupil size on grating detection at various contrast levels. *Vision Res.* 15 645–648. 10.1016/0042-6989(75)90278-3 1138478

[B52] Woodhouse J. M. Campbell F. W. (1975). The role of the pupil light reflex in aiding adaptation to the dark. *Vision Res.* 15 649–653. 10.1016/0042-6989(75)90279-5 1138479

[B53] Wu Z. Su S. (2014). “Detection accuracy comparison between the high frequency and low frequency SSVEP-based BCIs,” in *Proceedings of the 2nd International Conference on Communications, Signal Processing, and Systems*, Tianjin, 307–312.

[B54] Xu M. Han J. Wang Y. Jung T. P. Ming D. (2020). Implementing over 100 command codes for a high-speed hybrid brain-computer interface using concurrent P300 and SSVEP features. *IEEE Trans. Biomed. Eng.* 67 3073–3082. 10.1109/TBME.2020.2975614 32149621

[B55] Yao Z. Ma X. Wang Y. Zhang X. Liu M. Pei W. (2018). High-Speed spelling in virtual reality with sequential hybrid BCIs. *IEICE Trans. Inform. Systems* 101 2859–2862. 10.1587/transinf.2018EDL8122 12739966

[B56] Zhu D. Bieger J. Molina G. G. Aarts R. M. (2010). A survey of stimulation methods used in SSVEP-based BCIs. *Comp. Intell. Neurosci.* 2010:702357. 10.1155/2010/702357 20224799 PMC2833411

