# Supplementary material for: A Hybrid Brain-Computer Interface Based on Visual Evoked Potential and Pupillary Response
Source: Front Hum Neurosci. 2022 Feb 3;16:834959. doi: 10.3389/fnhum.2022.834959 (PMC8850273; doi:10.3389/fnhum.2022.834959)
Supplement: Supplementary file 1 [file Data_Sheet_1.docx]

Supplementary Material

# Pre-experiment

## Methods

Since user experience was rarely reported in the previous studies, we designed a pre-experiment to compare the subjective perception of visual stimuli with different frequencies. As shown in Figure S1, 60 targets with flickering frequencies ranging from 1 to 60 Hz (with an interval of 1 Hz) were presented on a SAMSUNG monitor (resolution: 3840*1080 pixels, refresh rate: 240 Hz) in a random order. The size of each target was 120×120 pixels (3°×3°), while the distance between subject and monitor was 70cm.


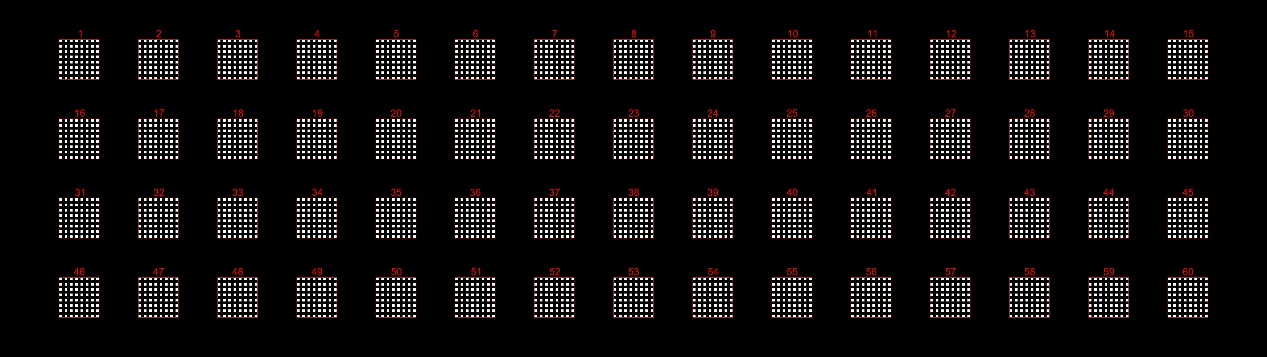


**Figure S1.** Stimulation interface of the 60-target pre-experiment.

Twelve subjects participated in this pre-experiment, of which 5 subjects participated in the h-BCI experiments. The pre-experiment consisted of 3 blocks. Each block contained 60 trials, corresponding to 60 targets cued in a random order. Each trial started with a visual cue (a red triangle) that appeared below the target for 1s, and the subjects were asked to fixate at the target stimulus for the next 5s while all targets were flickering together. Then, the subjects completed the questionnaires (Bieger and Molina, 2010) about the comfort level (scores 1 to 5 correspond to very uncomfortable, uncomfortable but tolerable, somewhat uncomfortable, comfortable, very comfortable, respectively), the perception of flicker (scores 1 to 5 correspond to very strong flicker, strong flicker, slight flicker, perceptible flicker, imperceptible flicker, respectively) and the preference level (score 1 indicates the worst and 5 indicates the best) for the cued target.

## Results

Figure S2 showed the average scores across 12 subjects and 3 blocks. One-way repeated-measures analysis of variance (RMANOVA) indicated that the scores in the low frequency (e.g., 1 Hz, comfort level: 3.42±0.35, preference level: 3.08±0.35) were comparable (comfort level: F(1,11)=0.60, p>0.05; preference level: F(1,11)=1.06, p>0.05) to the high frequency (e.g., 30 Hz, comfort level: 3.67±0.15, preference level: 3.33±0.15), although the low frequency had a stronger sense of flicker (low frequency: 2.31±0.22, high frequency: 3.42±0.15, F(1,11)=20.76, p<0.05). Meanwhile, both the two frequencies had a significantly better user experience than the alpha frequency range (e.g., 10 Hz, comfort level: 1.56±0.17, perception of flicker: 1.25±0.11, preference level: 1.36±0.13, p<0.05).

**Figure S2.** The user experience scores of the pre-experiment averaged across 12 subjects and 3 blocks. The error bars represent the standard errors.
